# Prevalence of and factors associated with multiple neurodevelopmental disorders among children and adolescents: a nationwide cross-sectional study

**DOI:** 10.3389/fpubh.2026.1875185

**Published:** 2026-09-11

**Authors:** Longshuang Han, Peixin Han, Jinfeng Ding, Lanting Yang, Zhongyuan Xu, Jiang Long, Patricia M. Davidson, Jialing Lin

**Affiliations:** 1 School of Law, Central South University, Changsha, Hunan, China; 2 Faculty of Science and Technology, Beijing Normal-Hong Kong Baptist University, Zhuhai, Guangdong, China; 3 Xiangya School of Nursing, Central South University, Changsha, Hunan, China; 4 School of Global and Population Health, The University of Western Australia, Perth, WA, Australia; 5 Division of Clinical Pharmacy, Skaggs School of Pharmacy and Pharmaceutical Sciences, University of California, San Diego, CA, United States; 6 Shanghai Mental Health Center, Shanghai Jiao Tong University School of Medicine, Shanghai, China; 7 International Center for Future Health Systems, University of New South Wales, Sydney, NSW, Australia

**Keywords:** children and adolescents, factors, multiple neurodevelopmental disorders, prevalence, risk management

## Abstract

**Objectives:**

Multiple neurodevelopmental disorders (NDDs) in children and adolescents are associated with greater impairment and reduced responsiveness to standard treatments, yet their prevalence and associated factors remain poorly understood. We aimed to investigate prevalence of, and factors associated with multiple NDDs among children and adolescents in the United States (US).

**Methods:**

This nationwide cross-sectional study used data from the National Survey of Children's Health 2016–2023. Multiple NDDs were defined as the concurrent presence of two or more conditions. Weighted prevalence estimates were calculated, and logistic regression was applied to assess associated factors of multiple NDDs adjusting for sociodemographic and health variables.

**Results:**

The prevalence of multiple NDDs among children aged 3–17 years increased from 5.8% in 2016 to 7.7% in 2023, with the strongest cross-sectional association observed for depression or anxiety (aOR = 5.53, 95% CI: 5.09–6.02), followed by fair or poor health (aOR = 2.82, 95% CI: 2.33–3.42), heart conditions (aOR = 1.93, 95% CI: 1.59–2.35), underweight status (aOR = 1.30, 95% CI: 1.13–1.50), and allergies (aOR = 1.17, 95% CI: 1.08–1.26). Lower odds were observed among female children (aOR = 0.40, 95% CI: 0.37–0.43) and those with higher parental education (aOR = 0.86, 95% CI: 0.80–0.93).

**Conclusion:**

This study revealed a rising prevalence of multiple NDDs and identified associated factors across clinical, demographic, and socioeconomic domains. Although the cross-sectional design precludes temporal or causal inference, the findings highlight the importance of integrated and equity-driven strategies to ensure timely access to health services and family-centered support for children and adolescents with multiple NDDs.

## 1 Introduction

Neurodevelopmental disorders (NDDs) are clinically and genetically heterogenous conditions that arise from atypical brain development or function and typically manifest in childhood and adolescence (1). In DSM-5 and ICD-11, commonly NDDs include attention-deficit/hyperactivity disorder (ADHD), autism spectrum disorder (ASD), learning disabilities, and speech disorders, while more severe but less prevalent conditions such as intellectual disability (ID) and Tourette syndrome are also encompassed within this definition group (2). We also included cerebral palsy in our NDD framework because it involves persistent motor impairment arising from disturbances in the developing brain and shows substantial clinical and genetic overlap with established NDDs (3). By impairing cognitive, social, and adaptive functioning, NDDs are associated with poorer health outcomes, increased healthcare utilization, and heightened mortality risk. They also pose substantial challenges for caregivers, clinicians, and health systems (4, 5).

NDDs frequently co-occur, with individuals exhibiting at least two conditions (i.e. multiple NDDs) being common (6, 7). It is reported that 14% of children and adolescents diagnosed with ADHD were also diagnosed with ASD in the United States (US) (8). Compared to single NDD, children experiencing multiple NDDs are more likely to have lower cognitive functions, poorer health-related quality of life, and experience severe disability and autoimmune diseases (9). They also show a greater tendency to be underweight or overweight/obese, report more serious life events, exhibit challenging and maladaptive behaviors, have poorer sleep quality, face increased psychosocial vulnerabilities, experience frequent anxiety and depressive symptoms, and suffer from gastrointestinal issues (10, 11). Moreover, multiple NDDs could be associated with not only greater impairment but also less responsiveness to standard treatments for either disorder (10).

Given the heterogeneity of clinical presentation and the involvement of interacting clinical, familial, and socioeconomic factors, research on prevalence and associated factors of multiple NDDs is essential (12). Recent evidence syntheses indicated substantial variation in prevalence estimates for single NDD across diagnostic categories and ascertainment methods, and NDDs have rarely been assessed as a whole (13). By contrast, pooled prevalence estimates are now available for specific conditions, including ADHD in children and adolescents (8.0%) and ASD in children (0.8%) (14, 15). While existing studies have largely focused on single NDD and identified various associated factors, such as age, sex, family adversity, lifestyle, biological functioning, prenatal and perinatal exposures, and socioeconomic status (16, 17), less is known about the factors associated with multiple NDDs. The mechanisms underlying the development of multiple co-occurring NDDs are considerably more complex, interrelated and multilevel, involving both genetic susceptibility and environmental determinants (18). Current evidence regarding factors associated with multiple NDDs remains limited, posing challenges for policymakers and clinicians in allocating resources and developing targeted support and service strategies for children with these complex conditions.

Therefore, leveraging data from the National Survey of Children's Health (NSCH), we aimed to estimate the prevalence of multiple NDDs and identify its associated factors among children and adolescents.

## 2 Methods

### 2.1 Data source and study population

This nationwide cross-sectional study used data from the NSCH 2016–2023. As these data are fully anonymized and publicly available, ethical approval was not required. The NSCH is a continuous, multistage, nationally representative survey of noninstitutionalized children in the US aged 0–17 years. Data were collected through mail and web-based surveys in English and Spanish with supports from the Maternal and Child Health Bureau, and National Center for Health Statistics across all 50 states and the District of Columbia (19). The overall response rates ranged from 35.8% to 43.1% (20).

The study sample contained children aged 3–17 years with complete data on NDDs. The age group was selected as the diagnosis of NDDs might not be accurate for children aged less than 3 years (21). As the analysis was based on an existing survey dataset, no separate sample size calculation was performed.

### 2.2 Outcome definitions and covariates

We defined multiple NDDs as the concurrent presence of two or more of the following NDDs: ADHD, ASD, learning disabilities, speech disorders, intellectual disability, cerebral palsy, and Tourette syndrome. This approach is line with similar measures of estimating multimorbidity (22). Information on NDDs was derived from parent or caregiver reported questions in NSCH. Children were considered to have a current NDD when the respondent reported that a doctor or other health care provider had ever told them that the child had cerebral palsy, Tourette syndrome, ASD, or ADHD, also reported that the child currently had the condition. Intellectual disability, speech or other language disorder, and learning disability were defined using the corresponding NSCH questions referring to a doctor, other health care provider, or educator, followed by a question on current status (23).

Covariates included a range of individual- and household-level characteristics. Individual-level variables were assessed at the time of the survey and included age, sex, race/ethnicity, country of birth, and current health conditions (e.g., allergies, asthma, heart conditions, diabetes, depression, or anxiety). Depression and anxiety were assessed using separate NSCH items. We constructed a composite binary variable, “depression or anxiety,” which was coded as present when the caregiver reported that the child currently had depression, anxiety, or both, and as absent when neither current condition was reported. Additional individual-level measures included body mass index (BMI), physical activity in the past week, and healthcare utilization in the past 12 months (including visits to healthcare providers, specialists, or dental check-ups). Adverse childhood experiences (ACEs), such as parental divorce, death, or imprisonment, exposure to violence, living with someone experiencing mental illness, suicidal behavior, severe depression, or substance use were also considered.

Household characteristics included family structure, household income, health insurance coverage, the highest level of education attained within the household, primary language spoken at home, and whether the residence was in a metropolitan principal city.

### 2.3 Statistical analysis

Descriptive analyses were first conducted to present the characteristics of the full set of variables among the children included in the study. Continuous variables were summarized as means with standard deviations (SDs), while categorical variables were reported as frequencies and percentages. The prevalence of multiple NDDs was estimated using weighted analyses to account for the survey's sampling design and to ensure the representation of children (20). To examine associated factors with the prevalence of multiple NDDs, univariate and multivariate logistic regression models adjusting for all individual and household characteristics were employed. The strength of association was presented as odds ratios (ORs) with 95% confidence intervals (CIs). Variance inflation factors (VIFs) were used to assess multicollinearity among associated variables. Variables with VIF values exceeding 10 (VIF>10) were considered to exhibit multicollinearity and were excluded from the final models. All analyses were performed using R version 4.5.0 (R Core Team 2017, Vienna, Austria). Given the large number of univariate comparisons, these analyses were considered exploratory and were interpreted cautiously. Greater emphasis was based on the multivariable logistic regression results, which accounted for potential confounding. Missing covariate values were retained as a separate “Unknown” category in the primary analyses. This approach was selected to preserve the full analytical sample and avoid substantial loss of precision given the high proportion of missing values for several variables. No complete-case analysis or multiple imputation was performed. All analyses accounted for the complex survey design of the NSCH by incorporating survey weights, strata, and household units. When pooling the 2016–2023 survey cycles, the annual survey weights were rescaled by dividing each year's weight by the number of pooled survey years, following recommended procedures for pooled cross-sectional analyses (24).

### 2.4 Sensitivity analysis

In addition to our primary outcome of multiple NDDs (≥2 NDDs), we conducted a sensitivity analysis with defining ‘complex NDDs' as the presence of three or more NDDs (6). This threshold was chosen to identify a subgroup with higher clinical complexity, to assess whether the identified associated variable remained consistent or changed in association with greater diagnostic prevalence. Univariate and multivariate logistic regression models were performed to test the factors associated with the presence of complex NDDs.

## 3 Results

### 3.1 Characteristics of study participants

Of the 277,792 children aged 3–17 years surveyed during 2016 and 2023 in the study (Supplementary Figure 1), the mean age was 10.3 years (SD: 4.5), and 51.7% were male (Table 1). Two-thirds were non-Hispanic white (67.0%), and most were US-born (95.8%). Reported health conditions included allergies (24.3%), overweight (8.8%), depression or anxiety (12.1%), and asthma (8.1%), while less common conditions included heart disease (1.4%) and diabetes (0.4%). Only 16.0% reported daily exercise in the past week.

**Table 1:** Characteristics of participants by multiple neurodevelopmental disorders (NDDs) status.

Characteristics	Overall	No	Yes	*P*-value
Total	277,792 (100.0)	258,159 (100.0)	19,633 (100.0)	
Survey year, n (%)				< 0.001
2016	42,108 (15.2)	39,495 (15.3)	2,613 (13.3)	
2017	18,251 (6.6)	17,030 (6.6)	1,221 (6.2)	
2018	25,854 (9.3)	24,192 (9.4)	1,662 (8.5)	
2019	25,364 (9.1)	23,648 (9.2)	1,716 (8.7)	
2020	36,659 (13.2)	34,103 (13.2)	2,556 (13.0)	
2021	41,074 (14.8)	38,086 (14.8)	2,988 (15.2)	
2022	44,179 (15.9)	40,773 (15.8)	3,406 (17.3)	
16.3-7.2,-1.3498.5pt 2023	44,303 (15.9)	40,832 (15.8)	3,471 (17.7)	
Individual characteristics				
Age (years), mean (standard deviation)	10.3 (4.5)	10.2 (4.6)	11.2 (4.1)	< 0.001
Sex, n (%)				< 0.001
Male	143,662 (51.7)	130,239 (50.4)	13,423 (68.4)	
Female	134,130 (48.3)	127,920 (49.6)	6,210 (31.6)	
Race/Ethnicity, n (%)				< 0.001
Non-Hispanic White	186,255 (67.0)	173,183 (67.1)	13,072 (66.6)	
Hispanic	36,499 (13.1)	33,834 (13.1)	2,665 (13.6)	
Non-Hispanic Black	17,643 (6.4)	16,033 (6.2)	1,610 (8.2)	
Asian	15,561 (5.6)	14,962 (5.8)	599 (3.1)	
Others	21,834 (7.9)	20,147 (7.8)	1,687 (8.6)	
Country of birth, n (%)				0.021
US	266,050 (95.8)	247,173 (95.7)	18,877 (96.1)	
Overseas	9,719 (3.5)	9,087 (3.5)	632 (3.2)	
Unknown	2,023 (0.7)	1,899 (0.7)	124 (0.6)	
Health condition, n (%)				< 0.001
Excellent/very good/good	273,828 (98.6)	255,575 (99.0)	18,253 (93.0)	
Fair/poor	3,565 (1.3)	2,225 (0.9)	1,340 (6.8)	
Unknown	399 (0.1)	359 (0.1)	40 (0.2)	
Body mass index				< 0.001
Healthy weight	107,819 (38.8)	100,212 (38.8)	7,607 (38.7)	
Underweight	11,995 (4.3)	10,792 (4.2)	1,203 (6.1)	
Overweight	24,368 (8.8)	22,257 (8.6)	2,111 (10.8)	
Obese	23,509 (8.5)	20,737 (8.0)	2,772 (14.1)	
Unknown	110,101 (39.6)	104,161 (40.3)	5,940 (30.3)	
Exercise everyday in the past week, n (%)				< 0.001
No	169,778 (61.1)	155,865 (60.4)	13,913 (70.9)	
Yes	44,547 (16.0)	41,337 (16.0)	3,210 (16.4)	
Unknown	63,467 (22.8)	60,957 (23.6)	2,510 (12.8)	
Healthcare provider visit in the past year, n (%)				< 0.001
No	41,920 (15.1)	40,197 (15.6)	1,723 (8.8)	
Yes	235,506 (84.8)	217,610 (84.3)	17,896 (91.2)	
Unknown	366 (0.1)	352 (0.1)	14 (0.1)	
Specialist visit in the past year, n (%)				< 0.001
No	232,224 (83.6)	219,338 (85.0)	12,886 (65.6)	
Yes	43,826 (15.8)	37,197 (14.4)	6,629 (33.8)	
Unknown	1,742 (0.6)	1,624 (0.6)	118 (0.6)	
Emergency department visit in the past year, n (%)				< 0.001
No	235,659 (84.8)	220,644 (85.5)	15,015 (76.5)	
Yes	41,070 (14.8)	36,519 (14.1)	4,551 (23.2)	
Unknown	1,063 (0.4)	996 (0.4)	67 (0.3)	
Treatment or counseling from a mental health professional in the past year, n (%)				< 0.001
No	240,610 (86.6)	229,468 (88.9)	11,142 (56.8)	
Yes	35,991 (13.0)	27,590 (10.7)	8,401 (42.8)	
Unknown	1,191 (0.4)	1,101 (0.4)	90 (0.5)	
Dental check-up in the past year, n (%)				< 0.001
No	15,800 (5.7)	14,796 (5.7)	1,004 (5.1)	
Yes	225,506 (81.2)	209,807 (81.3)	15,699 (80.0)	
Unknown	36,486 (13.1)	33,556 (13.0)	2,930 (14.9)	
Ever experienced parent/guardian divorced, n (%)				< 0.001
No	205,348 (73.9)	192,979 (74.8)	12,369 (63.0)	
Yes	63,681 (22.9)	56,979 (22.1)	6,702 (34.1)	
Unknown	8,763 (3.2)	8,201 (3.2)	562 (2.9)	
Ever experienced parent/guardian died, n (%)				< 0.001
No	260,022 (93.6)	242,065 (93.8)	17,957 (91.5)	
Yes	8,578 (3.1)	7,498 (2.9)	1,080 (5.5)	
Unknown	9,192 (3.3)	8,596 (3.3)	596 (3.0)	
Ever experienced parent/guardian served time in jail or prison, n (%)				< 0.001
No	251,343 (90.5)	234,742 (90.9)	16,601 (84.6)	
Yes	16,901 (6.1)	14,483 (5.6)	2,418 (12.3)	
Unknown	9,548 (3.4)	8,934 (3.5)	614 (3.1)	
Ever experienced being a victim of violence or witnessed violence in their neighborhood, n (%)				< 0.001
No	257,790 (92.8)	240,655 (93.2)	17,135 (87.3)	
Yes	10,211 (3.7)	8,379 (3.2)	1,832 (9.3)	
Unknown	9,791 (3.5)	9,125 (3.5)	666 (3.4)	
Ever experienced living with anyone who was mentally ill, suicidal, or severely depressed, n (%)				< 0.001
No	241,532 (86.9)	226,618 (87.8)	14,914 (76.0)	
Yes	26,244 (9.4)	22,181 (8.6)	4,063 (20.7)	
Unknown	10,016 (3.6)	9,360 (3.6)	656 (3.3)	
Ever experienced living with anyone who had a problem with alcohol or drugs, n (%)				< 0.001
No	241,215 (86.8)	225,619 (87.4)	15,596 (79.4)	
Yes	26,790 (9.6)	23,398 (9.1)	3,392 (17.3)	
Unknown	9,787 (3.5)	9,142 (3.5)	645 (3.3)	
Allergies, n (%)				< 0.001
No	209,832 (75.5)	197,172 (76.4)	12,660 (64.5)	
Yes	67,642 (24.3)	60,690 (23.5)	6,952 (35.4)	
Unknown	318 (0.1)	297 (0.1)	21 (0.1)	
Asthma, n (%)				< 0.001
No	253,463 (91.2)	236,671 (91.7)	16,792 (85.5)	
Yes	22,372 (8.1)	19,678 (7.6)	2,694 (13.7)	
Unknown	1,957 (0.7)	1,810 (0.7)	147 (0.7)	
Heart condition, n (%)				< 0.001
No	273,604 (98.5)	254,834 (98.7)	18,770 (95.6)	
Yes	3,841 (1.4)	2,995 (1.2)	846 (4.3)	
Unknown	347 (0.1)	330 (0.1)	17 (0.1)	
Diabetes, n (%)				< 0.001
No	276,198 (99.4)	256,727 (99.4)	19,471 (99.2)	
Yes	980 (0.4)	855 (0.3)	125 (0.6)	
Unknown	614 (0.2)	577 (0.2)	37 (0.2)	
Depression, n (%)				< 0.001
No	263,842 (95.0)	247,601 (95.9)	16,241 (82.7)	
Yes	13,271 (4.8)	9,924 (3.8)	3,347 (17.0)	
Unknown	679 (0.2)	634 (0.2)	45 (0.2)	
Anxiety, n (%)				< 0.001
No	245,889 (88.5)	234,299 (90.8)	11,590 (59.0)	
Yes	31,103 (11.2)	23,118 (9.0)	7,985 (40.7)	
Unknown	800 (0.3)	742 (0.3)	58 (0.3)	
Depression or anxiety, n (%)				< 0.001
No	242,925 (87.4)	231,740 (89.8)	11,185 (57.0)	
Yes	33,510 (12.1)	25,142 (9.7)	8,368 (42.6)	
16.3-7.2,-1.3498.5pt Unknown	1,357 (0.5)	1,277 (0.5)	80 (0.4)	
Household characteristics				
Family structure, n (%)				< 0.001
Two married parents	192,206 (69.2)	180,752 (70.0)	11,454 (58.3)	
Two unmarried parents	15,781 (5.7)	14,460 (5.6)	1,321 (6.7)	
Single parent	51,523 (18.5)	46,635 (18.1)	4,888 (24.9)	
Others	11,911 (4.3)	10,348 (4.0)	1,563 (8.0)	
Unknown	6,371 (2.3)	5,964 (2.3)	407 (2.1)	
Household income (federal poverty level), n (%)				< 0.001
< 1.0	32,839 (11.8)	29,331 (11.4)	3,508 (17.9)	
1.0-3.9	129,093 (46.5)	119,273 (46.2)	9,820 (50.0)	
4.0+	115,860 (41.7)	109,555 (42.4)	6,305 (32.1)	
Health insurance coverage, n (%)				< 0.001
Not insured	11,823 (4.3)	11,172 (4.3)	651 (3.3)	
Public	57,002 (20.5)	50,082 (19.4)	6,920 (35.2)	
Private	193,199 (69.5)	183,854 (71.2)	9,345 (47.6)	
Both	11,422 (4.1)	8,965 (3.5)	2,457 (12.5)	
Unspecified	458 (0.2)	432 (0.2)	26 (0.1)	
Unknown	3,888 (1.4)	3,654 (1.4)	234 (1.2)	
Highest education from the family, n (%)				< 0.001
Vocational (college) school or lower	104,854 (37.7)	95,450 (37.0)	9,404 (47.9)	
University graduate or higher	171,949 (61.9)	161,792 (62.7)	10,157 (51.7)	
Unknown	989 (0.4)	917 (0.4)	72 (0.4)	
Primary household spoken language, n (%)				< 0.001
English	256,295 (92.3)	237,611 (92.0)	18,684 (95.2)	
Spanish	10,210 (3.7)	9,704 (3.8)	506 (2.6)	
Others	9,820 (3.5)	9,488 (3.7)	332 (1.7)	
Unknown	1,467 (0.5)	1,356 (0.5)	111 (0.6)	
Residence in a metropolitan principal city, n (%)				0.052
No	146,281 (52.7)	135,879 (52.6)	10,402 (53.0)	
Yes	61,874 (22.3)	57,427 (22.2)	4,447 (22.7)	
Unknown	69,637 (25.1)	64,853 (25.1)	4,784 (24.4)	

Most children had a healthcare provider visit (84.8%) and a dental check-up (81.2%) within the past year. Visits to specialists and emergency departments were less frequent, at 15.8% and 14.8%, respectively. Nearly a quarter (22.9%) had experienced parental divorce; about one in ten lived with an individual who was mentally ill/severely depressed (9.4%) or had a problem with alcohol/drugs (9.6%). Fewer had experienced parental death (3.1%) or incarceration (6.1%) or had been a victim of or witnessed violence (3.7%).

Most children lived in a two-parent married household (69.2%), in families where at least one member had a university degree or higher (61.9%), and where English was the primary spoken language (92.3%). Most households fell into the 1.0–3.9 (46.5%) or 4.0+ (41.7%) federal poverty level brackets. The majority of participants were covered by private health insurance (69.5%), while 20.5% had public insurance and 4.3% were uninsured. Detailed characteristics are in Table 1.

### 3.2 Prevalence of multiple NDDs

**Figure 1:**
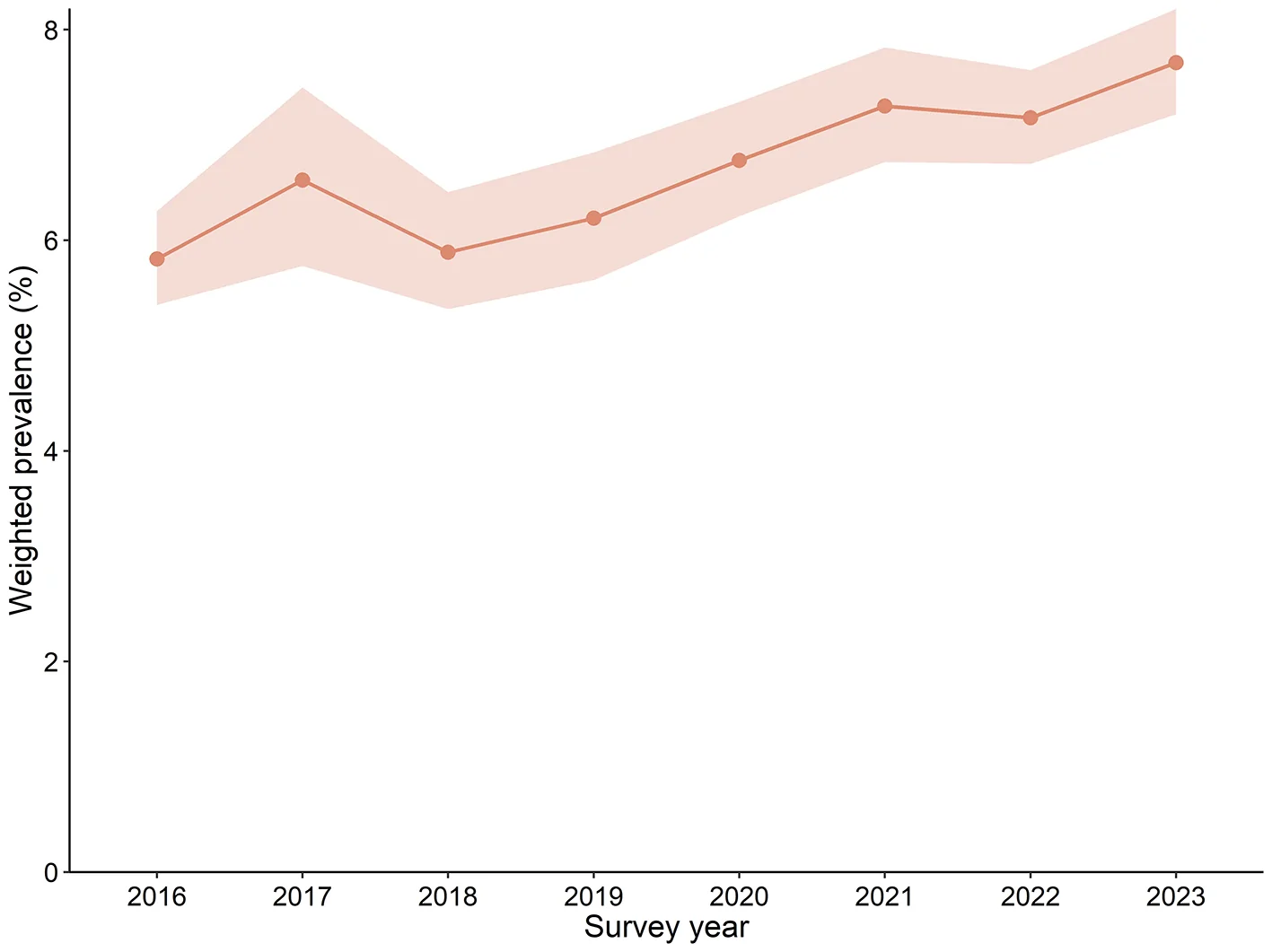
Prevalence of multiple neurodevelopmental disorders (NDDs) from 2016 to 2023.

**Figure 2:**
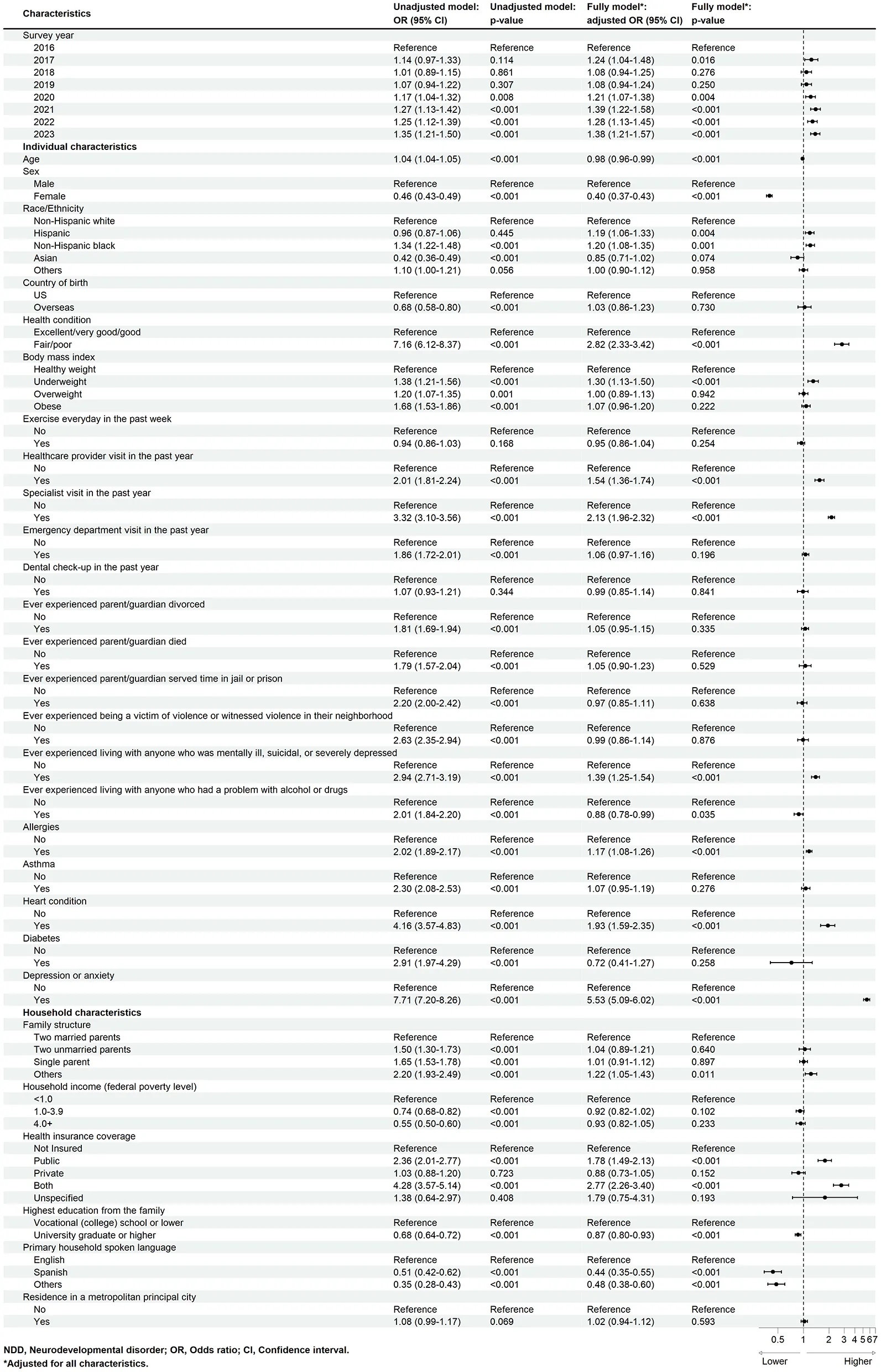
Factors associated with multiple neurodevelopmental disorders (NDDs).

The prevalence of multiple NDDs significantly increased from 5.8% in 2016 to 7.7% in 2023 (*P for trend* < 0.001) (Table 2 and Figures 1, 2). This prevalence was higher among males than females (8.9% vs. 4.3%, *P* < 0.001). The highest prevalence was observed in non-Hispanic Black children (8.7%) and the lowest in Asian children (2.9%) (*P* < 0.001).

**Table 2:** Prevalence of multiple neurodevelopmental disorders (NDDs) by characteristics.

Characteristics	No. population	No. multiple NDDs	Prevalence (95% CI)
Survey year			
2016	42,108	2,613	5.8 (5.4–6.3)
2017	18,251	1,221	6.6 (5.8–7.5)
2018	25,854	1,662	5.9 (5.3–6.5)
2019	25,364	1,716	6.2 (5.6–6.8)
2020	36,659	2,556	6.8 (6.2–7.3)
2021	41,074	2,988	7.3 (6.7–7.8)
2022	44,179	3,406	7.2 (6.7–7.6)
15.6-7.2,-1.3498.5pt2023	44,303	3,471	7.7 (7.2–8.2)
Individual characteristic			
Sex			
Male	143,662	13,423	8.9 (8.6–9.3)
Female	134,130	6,210	4.3 (4.1–4.5)
Race/Ethnicity			
Non–Hispanic White	186,255	13,072	6.6 (6.4–6.8)
Hispanic	36,499	2,665	6.4 (5.8–6.9)
Non–Hispanic Black	17,643	1,610	8.7 (8.0–9.4)
Asian	15,561	599	2.9 (2.5–3.3)
Others	21,834	1,687	7.2 (6.6–7.8)
Country of birth			
US	266,050	18,877	6.8 (6.6–7.0)
Overseas	9,719	632	4.7 (4.1–5.5)
Health condition			
Excellent/very good/good	273,828	18,253	6.2 (6.0–6.4)
Fair/poor	3,565	1,340	32.3 (29.0–35.7)
Body mass index			
Healthy weight	107,819	7,607	6.5 (6.2–6.8)
Underweight	11,995	1,203	8.7 (7.8–9.7)
Overweight	24,368	2,111	7.7 (7.0–8.4)
Obese	23,509	2,772	10.4 (9.7–11.3)
Exercise everyday in the past week			
No	169,778	13,913	7.5 (7.2–7.7)
Yes	44,547	3,210	7.0 (6.5–7.6)
Healthcare provider visit in the past year			
No	41,920	1,723	3.8 (3.4–4.2)
Yes	235,506	17,896	7.3 (7.1–7.6)
Specialist visit in the past year			
No	232,224	12,886	5.3 (5.1–5.5)
Yes	43,826	6,629	15.6 (14.9–16.4)
Emergency department visit in the past year			
No	235,659	15,015	5.9 (5.7–6.1)
Yes	41,070	4,551	10.5 (9.9–11.1)
Dental check–up in the past year			
No	15,800	1,004	6.2 (5.5–6.9)
Yes	225,506	15,699	6.5 (6.3–6.8)
Ever experienced parent/guardian divorced			
No	205,348	12,369	5.6 (5.4–5.9)
Yes	63,681	6,702	9.8 (9.3–10.3)
Ever experienced parent/guardian died			
No	260,022	17,957	6.5 (6.3–6.8)
Yes	8,578	1,080	11.2 (10.0–12.5)
Ever experienced parent/guardian served time in jail or prison			
No	251,343	16,601	6.2 (6.0–6.4)
Yes	16,901	2,418	12.7 (11.8–13.7)
Ever experienced being a victim of violence or witnessed violence in their neighborhood			
No	257,790	17,135	6.3 (6.1–6.5)
Yes	10,211	1,832	15.0 (13.7–16.4)
Ever experienced living with anyone who was mentally ill, suicidal, or severely depressed			
No	241,532	14,914	5.8 (5.6–6.0)
Yes	26,244	4,063	15.4 (14.5–16.4)
Ever experienced living with anyone who had a problem with alcohol or drugs			
No	241,215	15,596	6.2 (6.0–6.4)
Yes	26,790	3,392	11.7 (10.9–12.5)
Allergies			
No	209,832	12,660	5.6 (5.4–5.8)
Yes	67,642	6,952	10.7 (10.1–11.2)
Asthma			
No	253,463	16,792	6.1 (5.9–6.3)
Yes	22,372	2,694	13.0 (12.0–14.1)
Heart condition			
No	273,604	18,770	6.5 (6.3–6.7)
Yes	3,841	846	22.4 (19.9–25.0)
Diabetes			
No	276,198	19,471	6.6 (6.4–6.8)
Yes	980	125	17.1 (12.1–23.1)
Depression or anxiety			
No	242,925	11,185	4.5 (4.3–4.7)
15.6-7.2,-1.3498pt Yes	33,510	8,368	26.7 (25.7–27.8)
Household characteristics			
Family structure			
Two married parents	192,206	11,454	5.5 (5.3–5.7)
Two unmarried parents	15,781	1,321	8.0 (7.0–9.0)
Single parent	51,523	4,888	8.7 (8.3–9.2)
Others	11,911	1,563	11.3 (10.1–12.5)
Household income (federal poverty level)			
< 1.0	32,839	3,508	8.9 (8.3–9.6)
1.0–3.9	129,093	9,820	6.8 (6.5–7.1)
4.0+	115,860	6,305	5.1 (4.8–5.3)
Health insurance coverage			
Not insured	11,823	651	4.4 (3.8–5.1)
Public	57,002	6,920	9.9 (9.4–10.4)
Private	193,199	9,345	4.5 (4.4–4.7)
Both	11,422	2,457	16.6 (15.1–18.0)
Unspecified	458	26	6.0 (2.7–11.2)
Highest education from the family			
Vocational (college) school or lower	104,854	9,404	7.9 (7.5–8.2)
University graduate or higher	171,949	10,157	5.5 (5.3–5.7)
Primary household spoken language			
English	256,295	18,684	7.2 (7.0–7.4)
Spanish	10,210	506	3.8 (3.1–4.5)
Others	9,820	332	2.6 (2.1–3.2)
Residence in a metropolitan principal city			
No	146,281	10,402	6.5 (6.3–6.8)
Yes	61,874	4,447	7.0 (6.5–7.4)

OR, odds ratio; CI, confidence interval.

All six measured ACEs were linked to a higher prevalence (*P* < 0.001), with prevalences from 9.8% (parental divorce) to 15.4% (living with a household member with mental illness). Socioeconomic gradients were also observed. Prevalence was higher among children from single-parent families (8.7%) and other non-traditional family structures (11.3%) compared with those from two-married-parent families (5.5%) (all *P* < 0.001). Higher prevalence was also found among children from lower-income households (8.9% vs. 5.1% in higher-income households, *P* < 0.001), those with both public and private insurance (16.6%, *P* < 0.001), and those from families with lower educational attainment (7.9% vs. 5.5%, *P* < 0.001).

Children with fair or poor health had a higher prevalence (32.3% vs. 6.2%, *P* < 0.001), as did those who were underweight (8.7%), overweight (7.7%) or obese (10.4%) compared to those with a healthy weight (6.5%) (*P* < 0.001). Visits to a healthcare provider (7.3%), specialist (15.6%), and emergency department (10.5%) were all associated with higher prevalence (all *P* < 0.001). All co-occurring chronic conditions were associated with a higher prevalence, notably for depression or anxiety (26.7% vs. 4.5%, *P* < 0.001).

### 3.3 Associated factors with multiple NDDs

All factors had VIF values below 6.34 (most 1–2), indicating no substantial multicollinearity (Supplementary Table 3). After adjusting for all characteristics, the temporal increase in the prevalence of multiple NDDs remained a significant trend [2023 vs. 2016: adjusted OR (aOR) 1.38, 95%CI 1.21–1.57] (Figure 2). Children with depression or anxiety had over five times the odds of having multiple NDDs (aOR = 5.53, 95% CI: 5.09–6.02), followed by those with fair or poor health status (aOR = 2.82, 95% CI: 2.33–3.42), heart conditions (aOR = 1.93, 95% CI: 1.59–2.35), underweight status (aOR = 1.30, 95% CI: 1.13–1.50), and allergies (aOR = 1.17, 95% CI: 1.08–1.26), compared to children without these conditions. Frequent healthcare utilization, including specialist visits (aOR = 2.13, 95% CI: 1.96–2.32) and healthcare visits (aOR = 1.54, 95% CI: 1.36–1.74), was also associated with increased odds.

Several demographic characteristics were significant associated factors. Both Hispanic (aOR = 1.19, 95% CI: 1.06–1.33) and Non-Hispanic Black children (aOR = 1.20, 95% CI: 1.08–1.35) had higher odds of having multiple NDDs compared to non-Hispanic White children. Female sex was associated with lower odds (aOR = 0.40, 95% CI: 0.37–0.43).

Of the six measured ACEs, only living with a household member who had a mental illness remained a significant independent associated factor for multiple NDDs (aOR = 1.39, 95% CI: 1.25–1.54).

Socioeconomic disadvantage was also associated with increased prevalence of multiple NDDs. Children from nontraditional family structures (aOR = 1.22, 95% CI: 1.05–1.43) and those covered by public insurance (aOR = 1.78, 95% CI: 1.49–2.13) had significantly higher odds of multiple NDDs. Conversely, having parents with higher education (aOR = 0.87, 95% CI: 0.80–0.93) and speaking a language other than English at home (aOR = 0.48, 95% CI: 0.38–0.60), particularly Spanish (aOR = 0.44, 95% CI: 0.35–0.55), were associated with significantly lower odds.

### 3.4 Sensitivity analysis

Using ‘complex NDDs' as the outcome, prevalence was lower but showed a similar upward trend (2.2% in 2016 to 3.4% in 2023) (Supplementary Table 1 and Supplementary Figure 2). Key associated factors for complex NDDs were consistent with the primary analysis (male sex, fair/poor health, depression/anxiety). However, ACEs were not associated with complex NDDs in the adjusted model (Supplementary Table 1 and Supplementary Figure 3).

## 4 Discussion

Using data from the nationally representative 2016–2023 NSCH, we found an increasing trend in the prevalence of multiple NDDs among US children, rising from 5.8 % in 2016 to 7.7 % in 2023. Multiple NDDs were associated with a broad range of sociodemographic, clinical, and social factors. Of which, clinical factors were the strongest independent associations, such as depression or anxiety, fair or poor general health, underweight, comorbid physical conditions, and higher healthcare utilization. A number of demographic and socioeconomic factors were associated with multiple NDDs, particularly Hispanic or non-Hispanic Black ethnicity and parental mental illness. Associated factors included being female, higher household income, and higher parental education, may also reflect disparities in access to treatment sources.

Previous studies have reported a rising prevalence of single NDDs, particularly ASD and ADHD (21), however, few research considers NDDs collectively. Our study has filled this gap by demonstrating that the prevalence of multiple NDDs has increased significantly between 2016 and 2023, with a statistically marked acceleration after 2020. This trend aligns with Global Report on Children with Developmental Disabilities (25), which highlights that higher measured rates of ASD or ADHD in high Sociodemographic Index (SDI) settings may largely reflect better detection and service availability. Several explanations may account for this trend. First, broader diagnostic criteria, expansion of screening and diagnostic practices, improved awareness and acceptance of including NDDs as part of routine pediatric evaluations among parents, educators, and healthcare providers, have likely contributed to higher detection rates (26). Second, the acceleration observed after 2020 may reflect the profound impact of the COVID-19 pandemic on child development and mental health (27). However, whether this pattern reflects changes in underlying burden, ascertainment, healthcare access, or other pandemic-related contextual influences cannot be determined from cross-sectional data.

We found that a diagnosis of depression or anxiety was the strongest associated factor of multiple NDDs. While our study examined depression and anxiety as the associated factor, most studies have focused on the reverse direction on the impact of NDDs on comorbid anxiety and depression (28, 29). In this sense, depression, anxiety, and multiple NDDs appear to be cross-sectionally correlated, exerting reinforcing influences on one another. In line with the increased odds of multiple NDDs observed in our analysis, one possible explanation is the cumulative impact of adverse childhood experiences, which we also identified as a significant associated factor. Combined with maladaptive emotional regulation, these early-life stressors may help mediate the association between NDDs and depressive symptoms (30). Several other explanations may also contribute, including symptom overlap, shared vulnerability, and differential identification among children with greater clinical contact (31, 32). However, these possibilities cannot be examined, as the NSCH does not provide biomarker, genetic, or longitudinal data.

ACEs such as parental mental illness emerged as salient factors associated with increased prevalence of multiple NDDs, reflecting both environmental adversity and intergenerational vulnerability (33). This suggests the critical importance of family-centered support and integrated service that mitigate the impact of family adversity by engaging the entire family system rather than focusing solely on the child. Effective interventions developed for single NDDs, such as behavioral parent training, remain relevant for multiple NDDs when adapted to address family adversity and socio-emotional needs (34). Congruent with prior study (35), we also observed clear disparities by race and ethnicity, with Hispanic and non-Hispanic Black children facing higher risks, reflecting structural inequities in detection and access to appropriate care. The lower observed odds in households did not primarily speak English are more likely to reflect under identification and barriers to assessment than a true reduction in underlying burden. Similarly, the associations with higher parental education, stable family structure, and multilingual household contexts may reflect differences in social resources, healthcare access, and diagnostic recognition and should not be interpreted as direct protective effects. Children covered by public insurance were more frequently affected by NDDs. The concerning upward trend of multiple NDDs, combined with these deep-seated, multifaceted inequities, underscores the need for earlier identification, timelier referral for multidisciplinary assessment, and better care coordination for socioeconomically disadvantaged families, alongside culturally and linguistically appropriate services to improve access and continuity of care.

Fair or poor health status, heightened healthcare utilization, and specific physical comorbidities, including heart diseases and allergies, were also found to be an associated factor. Although shared inflammatory or immunological pathways have been proposed (36, 37), these mechanisms could not be assessed using the present data, and the observed associations may also reflect comorbidity or differential detection. Additionally, underweight status emerged as a significant clinical associated factor in our study. This may reflect atypical eating behaviors, such as feeding disturbances commonly observed in children with NDDs, which can lead to nutritional deficiencies and increased vulnerability to neurodevelopmental dysfunction (38). The nutritional challenges may be further exacerbated by low socioeconomic status (SES) due to limited access to balanced diets, reduced healthcare utilization, and heightened psychosocial stress (39). Notably, several associations were observed for depression/anxiety, health conditions, and healthcare utilization, all of which were assessed concurrently with NDDs. Simultaneous ascertainment also precludes establishing temporal order and may strengthen associations through symptom overlap or shared caregiver reporting. In addition, healthcare-utilization in the multivariable model may introduce potential collider-related bias if service use is influenced by both NDDs and other clinical or sociodemographic characteristics. These variables should therefore be interpreted as cross-sectional correlates of clinical co-occurrence and healthcare contact rather than as antecedent or causal predictors. Taking together, the pervasive impact of emotional, nutritional, and physical factors on multiple NDDs underscores the need for integrated pediatric, psychiatric, and subspecialty healthcare to ensure early detection, accurate diagnosis, and timely intervention.

Our study demonstrated that male sex was associated with 2.5-fold higher odds, consistent with previous finding by Stacy et al. (40). Sex-related differences in genetic susceptibility, clinical presentation, and diagnostic recognition, including the proposed female protective effect, may partly related to this pattern (41, 42). Males exhibit significantly greater genetic variability because of XY chromosomes, and possess a lower threshold for developing NDDs, conferring poorer resilience to risk exposures or impairments than females (43). Females typically showed higher empathy and lower systemizing, whereas many NDDs, such as ASD, are characterized by heightened systematic responses.

One of the strengths of this study is the large and representative sample. We investigated the prevalence of multiple NDDs and their associated factors, addressing a critical gap in existing literature that predominantly focused on single NDD and overlooked the clinical complexity of children with overlapping comorbidities. However, our study has several limitations. First, because we used cross-sectional data from NSCH, we were unable to identify causality, directionality, or temporality of the relationships. These findings should be interpreted cautiously, as temporal direction cannot be established in a cross-sectional study and the association may partly reflect diagnostic overlap or differential detection. Second, NSCH is based on caregiver recollection and is not independently verified, which may make these health data susceptible to subjective biases, recall inaccuracies and social desirability bias (44). Additionally, children may be at genetic risk of multiple NDDs, though detailed data from NSCH respondents regarding genetic risks was not available in this dataset. Third, the study period (2016–2023) spans the COVID-19 pandemic, potentially affecting the prevalence estimates and associated factors (45). Moreover, the severity of NDDs was not assessed, and residual confounding likely remains for contextual and socioeconomic factors, such as access to care and school environments. In addition, the multiple-NDD outcome was compositionally heterogeneous, encompassing different dyadic and higher-order combinations of conditions. Combination-specific prevalence and correlates were not examined in our analysis. A further limitation is that classifying missing covariate values as ‘Unknown' helped retain the full sample but may have introduced bias, particularly in the estimates for BMI and physical activity. Finally, participation in the NSCH is voluntary and limited to non-institutionalized households, potentially limiting the generalizability of our findings to more vulnerable or underserved populations.

## 5 Conclusions

This large cross-sectional study showed a concerning upward trend in the prevalence of multiple NDDs among children and adolescents between 2016 and 2023, and several clinical, demographic, and socioeconomic factors were associated with their occurrence. The identified clinical associated factors support consideration of shifting from disorder-specific approaches to integrated care models addressing psychiatric, nutritional, and physical problems. Observed disparities in the prevalence of multiple NDDs across demographic and socioeconomic groups further suggest the value of comprehensive, equity-driven strategies. Such strategies should mobilize healthcare, education, and community systems to ensure timely access to health services and family-centered support for affected children, adolescents, and their parents.

## Data Availability

The original contributions presented in the study are included in the article/Supplementary material, further inquiries can be directed to the corresponding author.
