# Supplementary material for: Prevalence of and factors associated with multiple neurodevelopmental disorders among children and adolescents: a nationwide cross-sectional study
Source: Front Public Health. 2026 Sep 11;14:1875185. doi: 10.3389/fpubh.2026.1875185 (PMC13612443; doi:10.3389/fpubh.2026.1875185)
Supplement: Supplementary file 1 [file Supplementary_File_1.docx]

**Supplementary Figures and Tables**

**Supplementary Table 1** Characteristics of participants by complex multiple neurodevelopmental disorders (NDDs) status

| **Characteristics** | **Overall** | **No** | **Yes** | **P-value** |
| --- | --- | --- | --- | --- |
| Total | 277,792 (100.0) | 277,169 (100.0) | 623 (100.0) |  |
| Survey year, n (%) |  |  |  | <0.001 |
| 2016 | 42,108 (15.2) | 42,014 (15.2) | 94 (15.1) |  |
| 2017 | 18,251 (6.6) | 18,209 (6.6) | 42 (6.7) |  |
| 2018 | 25,854 (9.3) | 25,803 (9.3) | 51 (8.2) |  |
| 2019 | 25,364 (9.1) | 25,304 (9.1) | 60 (9.6) |  |
| 2020 | 36,659 (13.2) | 36,579 (13.2) | 80 (12.8) |  |
| 2021 | 41,074 (14.8) | 40,981 (14.8) | 93 (14.9) |  |
| 2022 | 44,179 (15.9) | 44,071 (15.9) | 108 (17.3) |  |
| 2023 | 44,303 (15.9) | 44,208 (15.9) | 95 (15.2) |  |
| **Individual characteristics** |  |  |  |  |
| Age (years), mean (standard deviation) | 10.3 (4.5) | 10.3 (4.5) | 11.7 (3.9) | <0.001 |
| Sex, n (%) |  |  |  | <0.001 |
| Male | 143,662 (51.7) | 143,194 (51.7) | 468 (75.1) |  |
| Female | 134,130 (48.3) | 133,975 (48.3) | 155 (24.9) |  |
| Race/Ethnicity, n (%) |  |  |  | <0.001 |
| Non-Hispanic white | 186,255 (67.0) | 185,899 (67.1) | 356 (57.1) |  |
| Hispanic | 36,499 (13.1) | 36,390 (13.1) | 109 (17.5) |  |
| Non-Hispanic black | 17,643 (6.4) | 17,578 (6.3) | 65 (10.4) |  |
| Asian | 15,561 (5.6) | 15,512 (5.6) | 49 (7.9) |  |
| Others | 21,834 (7.9) | 21,790 (7.9) | 44 (7.1) |  |
| Country of birth, n (%) |  |  |  | <0.001 |
| US | 266,050 (95.8) | 265,476 (95.8) | 574 (92.1) |  |
| Overseas | 9,719 (3.5) | 9,675 (3.5) | 44 (7.1) |  |
| Unknown | 2,023 (0.7) | 2,018 (0.7) | 5 (0.8) |  |
| Health condition, n (%) |  |  |  | <0.001 |
| Excellent/very good/good | 273,828 (98.6) | 273,331 (98.6) | 497 (79.8) |  |
| Fair/poor | 3,565 (1.3) | 3,440 (1.2) | 125 (20.1) |  |
| Unknown | 399 (0.1) | 398 (0.1) | 1 (0.2) |  |
| Body mass index |  |  |  | <0.001 |
| Healthy weight | 107,819 (38.8) | 105,174 (39.0) | 2,645 (33.6) |  |
| Underweight | 11,995 (4.3) | 11,475 (4.3) | 520 (6.6) |  |
| Overweight | 24,368 (8.8) | 24,298 (8.8) | 70 (11.2) |  |
| Obese | 23,509 (8.5) | 23,400 (8.4) | 109 (17.5) |  |
| Unknown | 110,101 (39.6) | 109,931 (39.7) | 170 (27.3) |  |
| Exercise everyday in the past week, n (%) |  |  |  | <0.001 |
| No | 169,778 (61.1) | 169,337 (61.1) | 441 (70.8) |  |
| Yes | 44,547 (16.0) | 44,431 (16.0) | 116 (18.6) |  |
| Unknown | 63,467 (22.8) | 63,401 (22.9) | 66 (10.6) |  |
| Healthcare provider visit in the past year, n (%) |  |  |  | <0.001 |
| No | 41,920 (15.1) | 41,880 (15.1) | 40 (6.4) |  |
| Yes | 235,506 (84.8) | 234,923 (84.8) | 583 (93.6) |  |
| Unknown | 366 (0.1) | 366 (0.1) | 0 (0.0) |  |
| Specialist visit in the past year, n (%) |  |  |  | <0.001 |
| No | 232,224 (83.6) | 231,926 (83.7) | 298 (47.8) |  |
| Yes | 43,826 (15.8) | 43,505 (15.7) | 321 (51.5) |  |
| Unknown | 1,742 (0.6) | 1,738 (0.6) | 4 (0.6) |  |
| Emergency department visit in the past year, n (%) |  |  |  | <0.001 |
| No | 235,659 (84.8) | 235,237 (84.9) | 422 (67.7) |  |
| Yes | 41,070 (14.8) | 40,872 (14.7) | 198 (31.8) |  |
| Unknown | 1,063 (0.4) | 1,060 (0.4) | 3 (0.5) |  |
| Treatment or counseling from a mental health professional in the past year, n (%) |  |  |  | <0.001 |
| No | 240,610 (86.6) | 240,320 (86.7) | 290 (46.5) |  |
| Yes | 35,991 (13.0) | 35,661 (12.9) | 330 (53.0) |  |
| Unknown | 1,191 (0.4) | 1,188 (0.4) | 3 (0.5) |  |
| Dental check-up in the past year, n (%) |  |  |  | <0.001 |
| No | 15,800 (5.7) | 15,760 (5.7) | 40 (6.4) |  |
| Yes | 225,506 (81.2) | 225,041 (81.2) | 465 (74.6) |  |
| Unknown | 36,486 (13.1) | 36,368 (13.1) | 118 (18.9) |  |
| Ever experienced parent/guardian divorced, n (%) |  |  |  | <0.001 |
| No | 205,348 (73.9) | 204,937 (73.9) | 411 (66.0) |  |
| Yes | 63,681 (22.9) | 63,497 (22.9) | 184 (29.5) |  |
| Unknown | 8,763 (3.2) | 8,735 (3.2) | 28 (4.5) |  |
| Ever experienced parent/guardian died, n (%) |  |  |  | <0.001 |
| No | 260,022 (93.6) | 259,467 (93.6) | 555 (89.1) |  |
| Yes | 8,578 (3.1) | 8,537 (3.1) | 41 (6.6) |  |
| Unknown | 9,192 (3.3) | 9,165 (3.3) | 27 (4.3) |  |
| Ever experienced parent/guardian served time in jail or prison, n (%) |  |  |  | <0.001 |
| No | 251,343 (90.5) | 250,825 (90.5) | 518 (83.1) |  |
| Yes | 16,901 (6.1) | 16,820 (6.1) | 81 (13.0) |  |
| Unknown | 9,548 (3.4) | 9,524 (3.4) | 24 (3.9) |  |
| Ever experienced being a victim of violence or witnessed violence in their neighborhood, n (%) |  |  |  | <0.001 |
| No | 257,790 (92.8) | 257,250 (92.8) | 540 (86.7) |  |
| Yes | 10,211 (3.7) | 10,154 (3.7) | 57 (9.1) |  |
| Unknown | 9,791 (3.5) | 9,765 (3.5) | 26 (4.2) |  |
| Ever experienced living with anyone who was mentally ill, suicidal, or severely depressed, n (%) |  |  |  | <0.001 |
| No | 241,532 (86.9) | 241,065 (87.0) | 467 (75.0) |  |
| Yes | 26,244 (9.4) | 26,117 (9.4) | 127 (20.4) |  |
| Unknown | 10,016 (3.6) | 9,987 (3.6) | 29 (4.7) |  |
| Ever experienced living with anyone who had a problem with alcohol or drugs, n (%) |  |  |  | <0.001 |
| No | 241,215 (86.8) | 240,712 (86.8) | 503 (80.7) |  |
| Yes | 26,790 (9.6) | 26,697 (9.6) | 93 (14.9) |  |
| Unknown | 9,787 (3.5) | 9,760 (3.5) | 27 (4.3) |  |
| Allergies, n (%) |  |  |  | <0.001 |
| No | 209,832 (75.5) | 209,497 (75.6) | 335 (53.8) |  |
| Yes | 67,642 (24.3) | 67,355 (24.3) | 287 (46.1) |  |
| Unknown | 318 (0.1) | 317 (0.1) | 1 (0.2) |  |
| Asthma, n (%) |  |  |  | <0.001 |
| No | 253,463 (91.2) | 252,950 (91.3) | 513 (82.3) |  |
| Yes | 22,372 (8.1) | 22,268 (8.0) | 104 (16.7) |  |
| Unknown | 1,957 (0.7) | 1,951 (0.7) | 6 (1.0) |  |
| Heart condition, n (%) |  |  |  | <0.001 |
| No | 273,604 (98.5) | 273,037 (98.5) | 567 (91.0) |  |
| Yes | 3,841 (1.4) | 3,785 (1.4) | 56 (9.0) |  |
| Unknown | 347 (0.1) | 347 (0.1) | 0 (0.0) |  |
| Diabetes, n (%) |  |  |  | <0.001 |
| No | 276,198 (99.4) | 275,591 (99.4) | 607 (97.4) |  |
| Yes | 980 (0.4) | 965 (0.3) | 15 (2.4) |  |
| Unknown | 614 (0.2) | 613 (0.2) | 1 (0.2) |  |
| Depression, n (%) |  |  |  | <0.001 |
| No | 263,842 (95.0) | 263,344 (95.0) | 498 (79.9) |  |
| Yes | 13,271 (4.8) | 13,148 (4.7) | 123 (19.7) |  |
| Unknown | 679 (0.2) | 677 (0.2) | 2 (0.3) |  |
| Anxiety, n (%) |  |  |  | <0.001 |
| No | 245,889 (88.5) | 245,643 (88.6) | 246 (39.5) |  |
| Yes | 31,103 (11.2) | 30,730 (11.1) | 373 (59.9) |  |
| Unknown | 800 (0.3) | 796 (0.3) | 4 (0.6) |  |
| Depression or anxiety, n (%) |  |  |  | <0.001 |
| No | 242,925 (87.4) | 242,689 (87.6) | 236 (37.9) |  |
| Yes | 33,510 (12.1) | 33,128 (12.0) | 382 (61.3) |  |
| Unknown | 1,357 (0.5) | 1,352 (0.5) | 5 (0.8) |  |
| **Household characteristics** |  |  |  |  |
| Family structure, n (%) |  |  |  | <0.001 |
| Two married parents | 192,206 (69.2) | 191,850 (69.2) | 356 (57.1) |  |
| Two unmarried parents | 15,781 (5.7) | 15,746 (5.7) | 35 (5.6) |  |
| Single parent | 51,523 (18.5) | 51,356 (18.5) | 167 (26.8) |  |
| Others | 11,911 (4.3) | 11,863 (4.3) | 48 (7.7) |  |
| Unknown | 6,371 (2.3) | 6,354 (2.3) | 17 (2.7) |  |
| Household income (federal poverty level), n (%) |  |  |  | <0.001 |
| <1.0 | 32,839 (11.8) | 32,704 (11.8) | 135 (21.7) |  |
| 1.0-3.9 | 129,093 (46.5) | 128,768 (46.5) | 325 (52.2) |  |
| 4.0+ | 115,860 (41.7) | 115,697 (41.7) | 163 (26.2) |  |
| Health insurance coverage, n (%) |  |  |  | <0.001 |
| Not Insured | 11,823 (4.3) | 11,576 (4.3) | 247 (3.1) |  |
| Public | 57,002 (20.5) | 53,864 (20.0) | 3,138 (39.9) |  |
| Private | 193,199 (69.5) | 190,226 (70.5) | 2,973 (37.8) |  |
| Both | 11,422 (4.1) | 10,010 (3.7) | 1,412 (17.9) |  |
| Unspecified | 458 (0.2) | 447 (0.2) | 11 (0.1) |  |
| Unknown | 3,888 (1.4) | 3,798 (1.4) | 90 (1.1) |  |
| Highest education from the family, n (%) |  |  |  | <0.001 |
| Vocational (college) school or lower | 104,854 (37.7) | 104,532 (37.7) | 322 (51.7) |  |
| University graduate or higher | 171,949 (61.9) | 171,650 (61.9) | 299 (48.0) |  |
| Unknown | 989 (0.4) | 987 (0.4) | 2 (0.3) |  |
| Primary household spoken language, n (%) |  |  |  | 0.001 |
| English | 256,295 (92.3) | 255,744 (92.3) | 551 (88.4) |  |
| Spanish | 10,210 (3.7) | 10,173 (3.7) | 37 (5.9) |  |
| Others | 9,820 (3.5) | 9,792 (3.5) | 28 (4.5) |  |
| Unknown | 1,467 (0.5) | 1,460 (0.5) | 7 (1.1) |  |
| Residence in a metropolitan principal city, n (%) |  |  |  | 0.233 |
| No | 146,281 (52.7) | 142,062 (52.6) | 4,219 (53.6) |  |
| Yes | 61,874 (22.3) | 60,041 (22.2) | 1,833 (23.3) |  |
| Unknown | 69,637 (25.1) | 67,818 (25.1) | 1,819 (23.1) |  |

**Supplementary Table 2** Prevalence of complex multiple neurodevelopmental disorders (NDDs) by characteristics

| **Characteristics** | **No.**  **population** | **No.**  **complex NDDs** | **Prevalence (95% CI)** |
| --- | --- | --- | --- |
| Survey year |  |  |  |
| 2016 | 42,108 | 1,007 | 2.2 (2.0-2.5) |
| 2017 | 18,251 | 475 | 2.9 (2.3-3.6) |
| 2018 | 25,854 | 671 | 2.8 (2.3-3.2) |
| 2019 | 25,364 | 659 | 2.5 (2.1-2.9) |
| 2020 | 36,659 | 1,029 | 2.8 (2.4-3.1) |
| 2021 | 41,074 | 1,187 | 3.0 (2.6-3.4) |
| 2022 | 44,179 | 1,392 | 3.0 (2.7-3.4) |
| 2023 | 44,303 | 1,451 | 3.4 (3.1-3.8) |
| **Individual characteristics** |  |  |  |
| Sex |  |  |  |
| Male | 143,662 | 5,663 | 4.0 (3.8-4.3) |
| Female | 134,130 | 2,208 | 1.6 (1.4-1.7) |
| Race/Ethnicity |  |  |  |
| Non-Hispanic white | 186,255 | 4,990 | 2.5 (2.4-2.7) |
| Hispanic | 36,499 | 1,144 | 3.0 (2.6-3.5) |
| Non-Hispanic black | 17,643 | 734 | 4.1 (3.6-4.6) |
| Asian | 15,561 | 325 | 1.6 (1.3-1.9) |
| Others | 21,834 | 678 | 2.7 (2.4-3.1) |
| Country of birth |  |  |  |
| US | 266,050 | 7,512 | 2.8 (2.7-3.0) |
| Overseas | 9,719 | 309 | 2.8 (2.2-3.5) |
| Health condition |  |  |  |
| Excellent/very good/good | 273,828 | 7,033 | 2.5 (2.4-2.7) |
| Fair/poor | 3,565 | 822 | 19.8 (17.3-22.5) |
| Body mass index |  |  |  |
| Healthy weight | 107,819 | 2,645 | 2.4 (2.2-2.6) |
| Underweight | 11,995 | 520 | 3.9 (3.3-4.6) |
| Overweight | 24,368 | 808 | 3.1 (2.6-3.7) |
| Obese | 23,509 | 1,171 | 4.2 (3.8-4.7) |
| Exercise everyday in the past week |  |  |  |
| No | 169,778 | 5,464 | 3.1 (2.9-3.3) |
| Yes | 44,547 | 1,197 | 2.8 (2.4-3.2) |
| Healthcare provider visit in the past year |  |  |  |
| No | 41,920 | 605 | 1.6 (1.3-1.8) |
| Yes | 235,506 | 7,264 | 3.1 (3.0-3.3) |
| Specialist visit in the past year |  |  |  |
| No | 232,224 | 4,630 | 2.0 (1.9-2.2) |
| Yes | 43,826 | 3,196 | 8.0 (7.4-8.6) |
| Emergency department visit in the past year |  |  |  |
| No | 235,659 | 5,860 | 2.4 (2.3-2.6) |
| Yes | 41,070 | 1,985 | 4.8 (4.3-5.3) |
| Dental check-up in the past year |  |  |  |
| No | 15,800 | 425 | 2.8 (2.3-3.4) |
| Yes | 225,506 | 6,082 | 2.6 (2.5-2.8) |
| Ever experienced parent/guardian divorced |  |  |  |
| No | 205,348 | 5,120 | 2.5 (2.3-2.6) |
| Yes | 63,681 | 2,513 | 4.0 (3.6-4.3) |
| Ever experienced parent/guardian died |  |  |  |
| No | 260,022 | 7,197 | 2.8 (2.7-3.0) |
| Yes | 8,578 | 427 | 4.7 (3.8-5.7) |
| Ever experienced parent/guardian served time in jail or prison |  |  |  |
| No | 251,343 | 6,703 | 2.7 (2.6-2.9) |
| Yes | 16,901 | 919 | 4.7 (4.2-5.2) |
| Ever experienced being a victim of violence or witnessed violence in their neighborhood |  |  |  |
| No | 257,790 | 6,872 | 2.7 (2.5-2.9) |
| Yes | 10,211 | 711 | 6.0 (5.2-6.8) |
| Ever experienced living with anyone who was mentally ill, suicidal, or severely depressed |  |  |  |
| No | 241,532 | 5,977 | 2.5 (2.4-2.7) |
| Yes | 26,244 | 1,615 | 6.5 (5.8-7.2) |
| Ever experienced living with anyone who had a problem with alcohol or drugs |  |  |  |
| No | 241,215 | 6,377 | 2.7 (2.6-2.9) |
| Yes | 26,790 | 1,225 | 4.3 (3.8-4.8) |
| Allergies |  |  |  |
| No | 209,832 | 4,936 | 2.3 (2.2-2.5) |
| Yes | 67,642 | 2,931 | 4.7 (4.3-5.1) |
| Asthma |  |  |  |
| No | 253,463 | 6,728 | 2.6 (2.4-2.7) |
| Yes | 22,372 | 1,089 | 5.4 (4.7-6.2) |
| Heart condition |  |  |  |
| No | 273,604 | 7,417 | 2.7 (2.6-2.9) |
| Yes | 3,841 | 446 | 12.8 (10.7-15.0) |
| Diabetes |  |  |  |
| No | 276,198 | 7,796 | 2.8 (2.7-2.9) |
| Yes | 980 | 65 | 10.6 (6.5-16.0) |
| Depression or anxiety |  |  |  |
| No | 242,925 | 4,235 | 1.8 (1.7-2.0) |
| Yes | 33,510 | 3,603 | 12.0 (11.2-12.8) |
| **Household characteristics** |  |  |  |
| Family structure |  |  |  |
| Two married parents | 192,206 | 4,541 | 2.3 (2.1-2.4) |
| Two unmarried parents | 15,781 | 528 | 3.6 (2.9-4.5) |
| Single parent | 51,523 | 2,011 | 3.8 (3.5-4.2) |
| Others | 11,911 | 630 | 4.8 (4.2-5.5) |
| Household income (federal poverty level) |  |  |  |
| <1.0 | 32,839 | 1,608 | 4.3 (3.8-4.8) |
| 1.0-3.9 | 129,093 | 4,007 | 2.9 (2.7-3.1) |
| 4.0+ | 115,860 | 2,256 | 1.8 (1.7-1.9) |
| Health insurance coverage |  |  |  |
| Not Insured | 11,823 | 247 | 1.7 (1.3-2.1) |
| Public | 57,002 | 3,138 | 4.6 (4.2-5.0) |
| Private | 193,199 | 2,973 | 1.6 (1.4-1.7) |
| Both | 11,422 | 1,412 | 9.3 (8.2-10.4) |
| Unspecified | 458 | 11 | 1.9 (0.6-4.2) |
| Highest education from the family |  |  |  |
| Vocational (college) school or lower | 104,854 | 3,942 | 3.5 (3.3-3.8) |
| University graduate or higher | 171,949 | 3,896 | 2.1 (2.0-2.3) |
| Primary household spoken language |  |  |  |
| English | 256,295 | 7,350 | 3.0 (2.8-3.1) |
| Spanish | 10,210 | 271 | 2.2 (1.7-2.9) |
| Others | 9,820 | 195 | 1.5 (1.1-1.9) |
| Residence in a metropolitan principal city |  |  |  |
| No | 146,281 | 4,219 | 2.7 (2.5-2.9) |
| Yes | 61,874 | 1,833 | 3.1 (2.8-3.5) |

OR, Odds ratio; CI, Confidence interval.

**Supplementary Table 3** Variance inflation factors (VIFs) for multiple and complex multiple neurodevelopmental disorders (NDDs) model by covariates

| **Variable** | **VIF** | **Tolerance** |
| --- | --- | --- |
| Survey year |  |  |
| 2017 | 1.357 | 0.737 |
| 2018 | 1.496 | 0.668 |
| 2019 | 1.494 | 0.669 |
| 2020 | 1.692 | 0.591 |
| 2021 | 1.791 | 0.558 |
| 2022 | 1.924 | 0.52 |
| 2023 | 1.927 | 0.519 |
| **Individual characteristics** |  |  |
| Age | 3.518 | 0.284 |
| Sex |  |  |
| Female | 1.016 | 0.984 |
| Race/Ethnicity |  |  |
| Hispanic | 1.404 | 0.712 |
| Non-Hispanic black | 1.147 | 0.872 |
| Asian | 1.333 | 0.75 |
| Others | 1.048 | 0.954 |
| Country of birth |  |  |
| US | 1.116 | 0.896 |
| Overseas | 1.129 | 0.886 |
| Health condition |  |  |
| Excellent/very good/good | 1.055 | 0.948 |
| Fair/poor | 1.002 | 0.998 |
| Body mass index |  |  |
| Underweight | 1.075 | 0.93 |
| Healthy weight | 1.13 | 0.885 |
| Overweight | 1.164 | 0.859 |
| Obese | 3.361 | 0.298 |
| Exercise everyday in the past week |  |  |
| Yes | 1.11 | 0.901 |
| Unknown | 2.366 | 0.423 |
| Healthcare provider visit in the past year |  |  |
| Yes | 1.141 | 0.877 |
| Unknown | 1.048 | 0.954 |
| Specialist visit in the past year |  |  |
| Yes | 1.125 | 0.889 |
| Unknown | 1.147 | 0.872 |
| Emergency department visit in the past year |  |  |
| Yes | 1.071 | 0.933 |
| Unknown | 1.187 | 0.843 |
| Dental check-up in the past year |  |  |
| Yes | 3.006 | 0.333 |
| Unknown | 2.948 | 0.339 |
| Ever experienced parent/guardian divorced |  |  |
| Yes | 1.756 | 0.57 |
| Unknown | 7.3 | 0.137 |
| Ever experienced parent/guardian died |  |  |
| Yes | 1.086 | 0.921 |
| Unknown | 6.333 | 0.158 |
| Ever experienced parent/guardian served time in jail or prison |  |  |
| Yes | 1.369 | 0.73 |
| Unknown | 5.69 | 0.176 |
| Ever experienced being a victim of violence or witnessed violence in their neighborhood |  |  |
| Yes | 1.163 | 0.86 |
| Unknown | 5.672 | 0.176 |
| Ever experienced living with anyone who was mentally ill, suicidal, or severely depressed |  |  |
| Yes | 1.251 | 0.799 |
| Unknown | 5.316 | 0.188 |
| Ever experienced living with anyone who had a problem with alcohol or drugs |  |  |
| Yes | 1.428 | 0.7 |
| Unknown | 5.675 | 0.176 |
| Allergies |  |  |
| Yes | 1.137 | 0.879 |
| Unknown | 1.004 | 0.996 |
| Asthma |  |  |
| Yes | 1.114 | 0.898 |
| Unknown | 1.005 | 0.995 |
| Heart condition |  |  |
| Yes | 1.021 | 0.979 |
| Unknown | 1.003 | 0.997 |
| Diabetes |  |  |
| Yes | 1.013 | 0.987 |
| Unknown | 1.004 | 0.996 |
| Depression or anxiety |  |  |
| Yes | 1.169 | 0.856 |
| Unknown | 1.005 | 0.995 |
| **Household characteristics** |  |  |
| Family structure |  |  |
| Two unmarried parents | 1.163 | 0.86 |
| Single parents | 1.718 | 0.582 |
| Others | 1.308 | 0.764 |
| Unknown | 2.262 | 0.442 |
| Household income (federal poverty level) |  |  |
| 1.0-3.9 | 3.006 | 0.333 |
| 4.0+ | 3.784 | 0.264 |
| Health insurance coverage |  |  |
| Public | 4.848 | 0.206 |
| Private | 5.794 | 0.173 |
| Both | 1.941 | 0.515 |
| Unspecified | 1.052 | 0.951 |
| Unknown | 1.386 | 0.722 |
| Highest education from the family |  |  |
| University graduate or higher | 1.426 | 0.701 |
| Unknown | 1.106 | 0.904 |
| Primary household spoken language |  |  |
| Spanish | 1.382 | 0.724 |
| Others | 1.291 | 0.774 |
| Unknown | 1.007 | 0.993 |
| Residence in a metropolitan principal city |  |  |
| Yes | 1.143 | 0.875 |
| Unknown | 1.185 | 0.844 |

Note: Shaded cells indicate VIF≥ 10, representing potential multicollinearity. Reference categories (e.g., Survey year 2016) are omitted from the table.

**Supplementary Figure 1** Selection of study participants

**Supplementary Figure 2** Prevalence of complex NDD s from 2016 to 2023

**Supplementary Figure 3** Factors associated with complex neurodevelopmental disorders (NDDs)
